# Test–retest reliability of multidimensional dyspnea profile recall ratings in the emergency department: a prospective, longitudinal study

**DOI:** 10.1186/1471-227X-12-6

**Published:** 2012-05-24

**Authors:** Mark B Parshall, Paula M Meek, David Sklar, Joe Alcock, Paula Bittner

**Affiliations:** 1 University of New Mexico College of Nursing, MSC 09–5350, 1 University of New Mexico, Albuquerque, NM 87131, USA; 2 College of Nursing, University of Colorado College of Nursing Denver, Education 2 North, 13120 East 19th Ave./Room 4115, P.O. Box 6511/C288-4, Aurora, CO 80045, USA; 3 University of New Mexico School of Medicine, Department of Emergency Medicine, MSC 10 5560, 1 University of New Mexico, Albuquerque, NM 87131, USA; 4 Emergency Medicine Service, Raymond G. Murphy VA Medical Center, 1501 San Pedro SE, Albuquerque, NM 87108, USA

**Keywords:** Dyspnea, Recall, Test–retest reliability, Questionnaires, Emergency department visits

## Abstract

**Background:**

Dyspnea is among the most common reasons for emergency department (ED) visits by patients with cardiopulmonary disease who are commonly asked to recall the symptoms that prompted them to come to the ED. The reliability of recalled dyspnea has not been systematically investigated in ED patients.

**Methods:**

Patients with chronic or acute cardiopulmonary conditions who came to the ED with dyspnea (*N* = 154) completed the Multidimensional Dyspnea Profile (MDP) several times during the visit and in a follow-up visit 4 to 6 weeks later (*n* = 68). The MDP has 12 items with numerical ratings of intensity, unpleasantness, sensory qualities, and emotions associated with how breathing felt when participants decided to come to the ED (recall MDP) or at the time of administration (“now” MDP). The recall MDP was administered twice in the ED and once during the follow-up visit. Principal components analysis (PCA) with varimax rotation was used to assess domain structure of the recall MDP. Internal consistency reliability was assessed with Cronbach’s alpha. Test–retest reliability was assessed with intraclass correlation coefficients (ICCs) for absolute agreement for individual items and domains.

**Results:**

PCA of the recall MDP was consistent with two domains (Immediate Perception, 7 items, Cronbach’s alpha = .89 to .94; Emotional Response, 5 items; Cronbach’s alpha = .81 to .85). Test–retest ICCs for the recall MDP during the ED visit ranged from .70 to .87 for individual items and were .93 and .94 for the Immediate Perception and Emotional Response domains. ICCs were much lower for the interval between the ED visit and follow-up, both for individual items (.28 to .66) and for the Immediate Perception and Emotional Response domains (.72 and .78, respectively).

**Conclusions:**

During an ED visit, recall MDP ratings of dyspnea at the time participants decided to seek care in the ED are reliable and sufficiently stable, both for individual items and the two domains, that a time lag between arrival and questionnaire administration does not critically affect recall of perceptual and emotional characteristics immediately prior to the visit. However, test–retest reliability of recall over a 4- to 6-week interval is poor for individual items and significantly attenuated for the two domains.

## Background

Recall of symptoms leading to an emergency department (ED) visit is a mainstay of clinical history-taking. Patients are commonly asked about symptoms or events prior to arrival that prompted the visit, but little is known about the reliability of recall self-reports, especially in relation to elapsed time. Accurate assessment of past symptoms is a key component of clinical decision making, including the choice of initial therapies, as well as consultation and hospitalization decisions. The reliability of symptom recall is also a potential concern in clinical research on symptoms in acute care settings because of time constraints on when patients can be approached, adequately informed of study purposes, and consented as research participants.

Several studies have examined test–retest reliability of self-reports of various symptoms during an emergency visit [1-4], although none involved recalling symptoms prior to the visit. For example, 100-mm visual analog scale pain ratings taken 1 minute apart were highly consistent (intraclass correlation coefficient [ICC] = .99) in a study of acute abdominal pain in ED patients [1]. Similarly, a study of acute pain in a pediatric ED showed high consistency in pain reports 1 to 3 hours apart using a 0-to-10 numerical rating scale in older children (≥ 8 years of age). The mean difference in pain ratings among those reporting no change was 0.2 scale points (95% confidence interval [CI]: 0.0, 0.4) [2]. By contrast, poor agreement was reported for repeated measures of descriptors of acute dizziness (e.g., spinning, unsteady, about to faint) in adult ED patients [3]. In another study, adult ED patients’ ratings of headache severity showed, at best, only moderate within-subjects agreement (κ = .51) and substantial within-subjects discordance (23%–38% of subjects) in responses to two semantically similar questions about present headache severity in relation to past history [4].

Symptom recall has been called “deceptively…complex” [5]. The reliability and validity of recall ratings depend on how patients are instructed, how many and which dimensions or characteristics they are asked to rate, and whether they are asked to recall a specific event, a particular interval (e.g., over the last 24 hours, week, or month) or some undefined usual state in relation to present discomfort [6].

Dyspnea is defined as “a subjective experience of breathing discomfort that consists of qualitatively distinct sensations that vary in intensity,” that involves “interactions among multiple physiological, psychological, social, and environmental factors, and may induce secondary physiological and behavioral responses” [7,8]. As a subjective experience, dyspnea is typically measured using various rating scales or questionnaires, many of which assess the impact of dyspnea on activity, functional status, or quality of life, rather than what breathing feels like [8]. Only a few of these instruments have been validated in ED patients [9,10].

In several studies conducted in EDs, dyspnea has been measured with a single-item rating such as a visual analog scale, numerical rating scale, or Borg scale [10-15]. A potential limitation of single-item scales is that unless instructions are clear about what aspect of the symptom to rate (e.g., how intense it is or how distressed one is by it) and consistently given, the symptom dimension being measured is potentially ambiguous [16]. In addition, the internal consistency reliability of single items is indeterminate (e.g., Cronbach’s coefficient alpha cannot be estimated for a single item) [17,18]. Although test–retest reliability of single items can be assessed, this can be challenging in the ED if what is being measured changes in response to acute treatment.

We are aware of only one study of dyspnea in an ED that assessed the test–retest reliability of recall ratings of dyspnea prior to a visit [19]. The median correlation for numeric ratings of seven dyspnea descriptors was .95, and the largest within-subjects difference for any descriptor was less than 1 point on a 0-to-10 numeric scale. However, that assessment was conducted with just a small subset (~10%) of the study sample, and conventional correlation coefficients are suboptimal for assessing test–retest reliability [18,20-22]. Results of that study [19] also suggested several potentially distinguishable dimensions of sensory quality in ED patients with chronic obstructive pulmonary disease (COPD), three of which (Smothering/Air hunger, Work/Effort, and Tightness) were confirmed in a subsequent study of hospital patients admitted for heart failure [23]. Only a few other studies have attempted to assess sensory qualities of dyspnea during ED visits [24,25]. The results of these studies suggest that multiple sensory quality dimensions of dyspnea may be common to patients of various diagnoses who come to an ED because of dyspnea. Although the clinical relevance of multiple dimensions of dyspnea in the acute care setting is not clearly established, in a study of ED patients with asthma, it was found that perceptions of increased work and effort in breathing persisted even after the sensation of tightness was relieved with albuterol [24].

The purpose of the present study was to assess the reliability of recall ratings of dyspnea in ED patients with acute or chronic pulmonary or cardiac disease. Specifically, we administered the Multidimensional Dyspnea Profile (MDP) [26-28] to obtain serial real-time and recall ratings during an ED visit and in an outpatient follow-up visit 4 to 6 weeks later. Results pertaining to the real-time ratings and overall psychometric performance of the MDP are being published separately [28].

## Methods

### Design, setting, and participants

The study had a prospective, longitudinal correlational design with repeated measures. The study was conducted in three urban EDs in the southwestern United States: at an academic health center, a Department of Veterans Affairs (VA) medical center, and a private, not-for-profit community hospital. The study was approved by the Human Research Review Committee of the University of New Mexico Health Sciences Center and the Raymond G. Murphy VA Medical Center Research and Development Committee, Albuquerque, NM. Signed, informed consent and Health Insurance Portability and Accountability Act (HIPAA) authorization were obtained from all participants. All recruitment and data collection were the responsibility of study personnel, who were credentialed by each facility in accordance with its research policies.

Patients who presented to the ED with breathing complaints due to acute or chronic pulmonary or cardiac conditions were potentially eligible. Exclusion criteria were: treatment for an acute coronary syndrome or advanced or metastatic cancer; absence of dyspnea at presentation; inability to speak or understand English; or previous participation in the study. Of 526 potentially eligible patients, 94 were discharged before recruitment could be completed. Of the remaining 432 patients, 182 (42%) agreed to participate.

### Measures

The MDP [26,28] was developed by an interdisciplinary team with expertise in respiratory physiology and psychophysics, pulmonary and critical care medicine, emergency medicine, acute care and emergency nursing, experimental psychology, and psychometrics to assess dimensions of dyspnea intensity, sensory quality, unpleasantness, and dyspnea-related affective distress. The instrument’s structure and content are based on a theoretical model of dyspnea sensation and affect [29] that was derived from an extensively validated multidimensional model of pain [30-36] that proposes potentially discriminable dimensions of sensation (intensity and quality) and two affective stages: immediate unpleasantness and emotional distress (e.g., judgments as to the meaning or significance of the experience). The relevance of this model to dyspnea is supported by multiple lines of laboratory and clinical research in dyspnea that have demonstrated the potential separability of dyspnea intensity and its associated emotional distress [37-44] or unpleasantness [26,45,46] as well as mechanistic distinctions among dyspnea sensory qualities (e.g., different peripheral afferent pathways or higher central nervous system processing) [19,25,42,47-58].

The MDP has a total of 12 items that use 0-to-10 numerical rating scales. Single items are used to rate the overall intensity of breathing sensation (0 = *No sensation*; 10 = *Maximum sensation*) and its unpleasantness (0 = *Neutral*; 10 = *Unbearable*). Five items measure the intensity of groupings of potentially distinguishable sensory qualities (0 = *None*; 10 = *As intense as I can imagine*):

· *My breathing requires muscle work or effort.*

· *I am not getting enough air, I feel hunger for air, or I am smothering.*

· *My breathing requires mental effort or concentration.*

· *My chest and lungs feel tight or constricted.*

· *I am breathing a lot (breathing rapidly, deeply, or heavily).*

(In the initial protocol, there was a single descriptor for *Work* or *Effort*; after enrollment of 27 patients, the MDP was amended to better distinguish between *muscle work or effort* and *mental effort or concentration*.) Five items measure emotions in relation to “how your breathing sensations make you feel”: *Depressed*, *Anxious*, *Frustrated*, *Angry*, and *Afraid* (0 = *None*; 10 = *The most I can imagine*).

We administered the MDP several times over the course of the ED visit, with questions referring to how breathing felt at that particular time (“now” wording) or how breathing felt at the time the participant decided to come to the ED (“recall” wording). Apart from the difference in time frame, the instructions and questions were identical.

Support for the potential independence of MDP ratings of intensity from unpleasantness and work/effort from air hunger have been reported in controlled physiological experiments in a laboratory setting [26]. However, principal components analysis of “now” ratings using the MDP in ED patients showed two components (domains) that jointly accounted for 66% to 74% of item variance [28]. The first domain comprised the single-item ratings of intensity and unpleasantness together with the five sensory quality ratings and was labeled Immediate Perception (7 items; Cronbach’s α > .90). The second domain consisted of the ratings of breathing-related emotional distress and was labeled Emotional Response (5 items; Cronbach’s α ≥ .84).

### Protocol

#### ED phase

Patients were triaged according to established departmental procedures. The initial contact for study participation took place after they had been evaluated and treatment was under way. Potentially eligible participants were identified by study staff, and the visit record was screened for excluding conditions. After ascertaining from the physician or registered nurse staff that the patient was sufficiently stable to be approached, potential participants were informed by ED personnel that a study was ongoing for which they might be eligible and given a brochure about the study prior to the initial contact by study staff. After the initial contact, those who expressed interest in participating were given a copy of the consent form and given time to read and consider it. After answering any questions, signed consent and full HIPAA authorization forms were obtained from all who agreed to participate.

As soon as possible after enrollment (Time 1), the study questionnaire was administered to assess how breathing felt at that time (“now” wording) and in a separate administration that asked participants to recall and rate how their breathing felt when they decided to come to the ED (“recall” wording: Time 0).

In the initial protocol, there was only a single administration of the Time 0 questionnaire (i.e., using the recall wording), but there were two subsequent administrations of the questionnaire using the “now” wording: an hour after the initial administration (Time 2) and, if possible, a third administration prior to leaving the department (Time 3). After 27 participants had been enrolled, a protocol amendment added a second recall administration immediately following the Time 2 administration of the “now” questionnaire. For the remainder of this report, the two recall administrations in the ED are referred to as Time 0a (at enrollment) and Time 0b (approximately an hour later). In general, the first questionnaire administration took no more than 5 minutes; subsequent administrations generally took less time.

#### Follow-up phase

As part of the consent process in the ED, potentially eligible persons were asked to indicate on the consent form whether they were willing to be contacted by study personnel at a later date to inquire about whether they might be willing to participate in a follow-up visit 4 to 6 weeks after the ED visit. Participation in the ED phase of the study was not conditional on whether or not they were willing to be contacted. Those who gave permission to be contacted for follow-up were invited to schedule an appointment. Participants with mobility or transportation issues were permitted to arrange a home visit if that was more convenient for them. The follow-up visit required a separate consent. The median (25^th^, 75^th^ percentile) time to the follow-up visit was 5 (4, 7) weeks. During the follow-up visit, participants completed several questionnaires, including a third recall administration of the MDP (Time 0c) to reassess how their breathing felt when they decided to come to the ED.

### Data analysis

Data were analyzed using IBM® SPSS® Statistics, version 19. Descriptive statistics included mean and standard deviation or median and percentiles for continuous variables and counts and percentages for categorical variables.

Principal components analysis with varimax rotation was used to assess the similarity of domains for the recall ratings to those previously reported for “now” ratings in the ED [28] (see Additional file 1 for details). Cronbach’s alpha was assessed for each domain at Times 0a, 0b, and 0c. A mean score (total of item scores/# of items) was calculated for each domain to standardize the domain score to the same 0-to-10 metric as the constituent items. Test–retest reliability of the recall ratings was assessed using two-way mixed-model ICCs for absolute agreement at the level of individual items (single measures ICC) and mean domain scores (average measures ICC).

Mean paired differences and 95% CIs for recall ratings were assessed graphically for individual items and domains across measurement intervals (Time 0a–Time 0b and Time 0a–Time 0c). Because item and domain scores were not normally distributed, Wilcoxon signed rank tests were calculated between Time 0a and 0b and between Time 0a and 0c for all items and the two domain scores. In addition, within-subjects differences between Times 0a–0b and 0a–0c were estimated at the 5^th^, 10^th^, 25^th^, 50^th^, 75^th^, 90^th^, and 95^th^ percentiles, and Hodges–Lehmann (nonparametric) estimates of median difference [59] with 95% CIs were calculated.

## Results

The sample consisted of 154 participants who were enrolled after the protocol amendment and for whom complete data were available on at least the Time 0a questionnaire. There were no significant differences in sex, age, race, or ethnicity between those who enrolled before versus after the protocol amendment.

The mean (SD) age of the sample was 53.2 (15.7) years; 45% (*n* = 70) were female; 78% were white, 7% were black, 5% were American Indian, and 10% were “other” or more than one race. Twenty-five per cent were Hispanic. Approximately 26% of participants (*n* = 41) had a diagnosis of COPD, 28% (*n* = 43) had asthma, 10% (*n* = 16) had heart failure, 16% (*n* = 25) had pneumonia, and 19% (*n* =29) had other cardiopulmonary diagnoses.

The component structure and domains for the MDP recall ratings were the same as reported previously [28] for the “now” ratings in these ED patients. For the three recall administrations, the Immediate Perception domain (7 items; Cronbach’s α = .89 to .94) and Emotional Response domain (5 items: Cronbach’s α = .81 to .85) jointly accounted for 63% to 71% of item variance (see Additional file 1: Table A1 — Principal components analysis).

Means, SDs, and quartiles for the MDP items and the two mean domain scores for each recall time period are shown in Table 1. Means for the Immediate Perception items were consistently higher than for the Emotional Response items in all three recall assessments (Table 1). The mean domain scores were approximately 2 scale points higher for the Immediate Perception domain compared with the Emotional Response domain in each recall assessment (Table 2). The Time 0a recall ratings and the concurrently obtained Time 1 “now” ratings were moderately and positively correlated for all items (Immediate Perception items: r = .30 to .45, *p* < .001; Emotional Response items: r = .46 to .60, *p* < .001) and domain scores (r = .42, *p* < .001 for Immediate Perception; r = .61, *p* < .001 for Emotional Response).

**Table 1:** Descriptive statistics for recall rating: how breathing felt “when you decided to come to the ED”

		**Emergency Department**								**Follow-up**			
		***Time 0a* (*N* = 154)**				***Time 0b* (*n* = 141–144)**				***Time 0c* (*n* = 67–68)**			
**Domain**	**Item (Dimension)**	**Mean (SD)**	**Percentiles**			**Mean (SD)**	**Percentiles**			**Mean (SD)**	**Percentiles**		
			**25**	**50**	**75**		**25**	**50**	**75**		**25**	**50**	**75**
**Immediate Perception**	Overall intensity	8.3 (2.1)	8.0	9.0	10.0	8.2 (2.1)	8.0	9.0	10.0	8.4 (1.8)	8.0	9.0	10.0
	Unpleasantness	8.5 (1.8)	8.0	9.0	10.0	8.1 (2.0)	7.0	9.0	10.0	8.5 (1.9)	8.0	9.0	10.0
	Muscle work/Effort	7.7 (2.7)	7.0	8.0	10.0	7.2 (3.0)	6.0	8.0	10.0	7.5 (2.7)	6.0	8.0	10.0
	Not enough air/Smothering/Air hunger	7.9 (2.5)	7.0	9.0	10.0	7.5 (2.9)	6.0	8.0	10.0	7.8 (2.6)	7.0	8.5	10.0
	Mental effort/Concentration	7.0 (3.4)	5.0	8.0	10.0	6.8 (3.3)	5.0	8.0	10.0	7.0 (3.2)	5.0	8.0	10.0
	Tight/Constricted	7.5 (3.0)	6.0	8.0	10.0	7.3 (3.0)	6.0	8.0	10.0	7.4 (3.0)	5.0	9.0	10.0
	Breathing a lot(rapid, deep, heavy)	7.4 (3.1)	6.0	9.0	10.0	7.1 (3.1)	5.0	8.0	10.0	6.6 (3.5)	3.3	8.0	10.0
	**Immediate Perception (7 item domain mean)**	7.8 (2.1)	6.7	8.3	9.6	7.5 (2.4)	6.2	8.1	9.4	7.6 (2.2)	6.1	8.0	9.4
**Emotional Response**	Depressed	4.4 (4.0)	0.0	5.0	9.0	4.3 (3.8)	0.0	4.0	8.0	5.0 (3.8)	0.0	5.0	8.0
	Anxious	6.5 (3.3)	4.0	8.0	9.0	6.1 (3.4)	3.0	7.0	9.0	7.0 (3.1)	5.0	8.0	9.0
	Frustrated	6.7 (3.4)	5.0	8.0	9.3	6.1 (3.4)	4.0	7.0	9.0	6.9 (3.3)	5.0	8.0	10.0
	Angry	4.5 (4.0)	0.0	5.0	8.0	4.0 (3.9)	0.0	3.0	8.0	4.4 (4.2)	0.0	3.0	9.0
	Afraid	6.0 (4.0)	2.0	8.0	10.0	5.6 (4.0)	1.0	7.0	10.0	5.5 (4.0)	0.0	7.0	9.0
	**Emotional Response (5 item domain mean)**	5.6 (2.9)	3.2	6.0	8.0	5.2 (2.9)	2.8	5.5	7.8	5.7 (2.9)	3.8	5.4	8.0

Time 0a: dyspnea at time of decision to come to ED recalled at enrollment during ED visit.

Time 0b: dyspnea at time of decision to come to ED recalled 1 hr after enrollment during ED visit.

Time 0c: dyspnea at time of decision to come to ED recalled at follow-up visit 4–6 weeks after ED visit.

**Table 2:** Within-subjects differences in mean scores for Immediate Perception vs. Emotional Response domains

				**95% CI**				
**Time**	**Mean Difference**^**a**^	**SD**	**SE**	**Lower**	**Upper**	***t***	**df**	**p <**
0a (N = 154)	2.1	2.52	0.20	1.74	2.54	10.55	153	0.001
0b (n = 142)	2.2	2.37	0.20	1.85	2.63	11.27	141	0.001
0c (n = 68)	1.8	2.47	0.30	1.22	2.42	6.07	67	0.001

^a^Positive within-subjects differences all indicate that mean domain scores for Immediate Perception > Emotional Response.

Time 0a: dyspnea at time of decision to come to ED recalled at enrollment during ED visit.

Time 0b: dyspnea at time of decision to come to ED recalled 1 hr after enrollment during ED visit.

Time 0c: dyspnea at time of decision to come to ED recalled at follow-up visit 4–6 weeks after ED visit.

Test–retest ICCs between recall ratings for the approximate 1-hour interval between Times 0a and 0b (Table 3) ranged from .69 to .86 for the individual items and .92 to .94 for the two domains. For the 4- to 6-week interval between the initial recall rating and the follow-up visit, ICCs were much lower (.28 to .66 for individual items and.72 to .78 for the two domains). Examination of 95% CIs around ICCs for the two test–retest intervals showed no overlap; therefore, all ICCs were significantly lower for the longer test–retest interval.

**Table 3:** Test–retest reliability of MDP recall ratings (single items and domains)

	**Time 0a–0b (*n* = 141–145)**			**Time 0a–0c(*n* = 67–68)**		
	**ICC**^**a**^	**95% CI**		**ICC**^**a**^	**95% CI**	
		**Lower**	**Upper**		**Lower**	**Upper**
***Immediate Perception*** items						
Intensity^b^	0.75	0.67	0.82	0.50	0.30	0.66
Unpleasantness^b^	0.80	0.72	0.86	0.55	0.36	0.70
Muscle work/Effort ^b^	0.77	0.69	0.83	0.45	0.24	0.62
Not enough air/Smothering/Air hunger^b^	0.80	0.73	0.86	0.43	0.21	0.60
Mental effort/Concentration^b^	0.78	0.71	0.84	0.53	0.34	0.68
Tight/Constricted^b^	0.73	0.65	0.80	0.35	0.12	0.54
Breathing a lot (rapid, deep, heavy)^b^	0.70	0.60	0.77	0.28	0.04	0.49
**Immediate Perception Domain (Mean of 7 items)**^**c**^	**0.93**	**0.90**	**0.95**	**0.72**	**0.54**	**0.83**
***Emotional Response*** items						
Depressed^b^	0.77	0.69	0.83	0.59	0.41	0.72
Anxious^b^	0.81	0.74	0.86	0.51	0.32	0.67
Frustrated^b^	0.70	0.60	0.78	0.47	0.26	0.63
Angry^b^	0.86	0.81	0.90	0.50	0.29	0.66
Afraid^b^	0.87	0.83	0.91	0.66	0.50	0.78
**Emotional Response Domain (Mean of 5 items)**^**c**^	**0.94**	**0.92**	**0.96**	**0.78**	**0.65**	**0.87**

^a^ICC = intraclass correlation coefficient: 2-way mixed ANOVA with subjects as random factor and test–retest interval as fixed factor.

^b^Single-measures, absolute agreement.

^c^Average measures, absolute agreement.

Time 0a: dyspnea at time of decision to come to ED recalled at enrollment during ED visit.

Time 0b: dyspnea at time of decision to come to ED recalled 1 hr after enrollment during ED visit.

Time 0c: dyspnea at time of decision to come to ED recalled at follow-up visit 4–6 weeks after ED visit.

**Figure 1:**
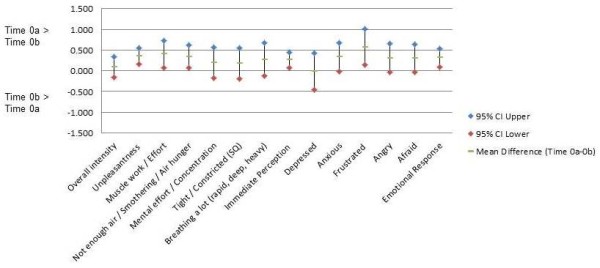
**Mean within-subjects differences (95% CI) Time 0a–0b (during ED visit) for individual items and subscales for Immediate Perception and Emotional Response (*n*** **= 141–145)**. Time 0a: time of decision to come to ED recalled at enrollment during ED visit. Time 0b: time of decision to come to ED recalled 1 hr after enrollment during ED visit.

For the test–retest interval during the ED visit (Time 0a to 0b; Figure 1), mean differences for individual items ranged from −0.02 to +0.57 points, with all but two falling between 0.1 and 0.5 points. The mean differences for the two mean domain scores were approximately +0.3 points for Immediate Perception and EmotionalResponse. All but one of the mean differences were positive, indicating that the second set of recall ratings in the ED (Time 0b) was consistently lower (less severe) than the initial ratings (Time 0a).

**Figure 2:**
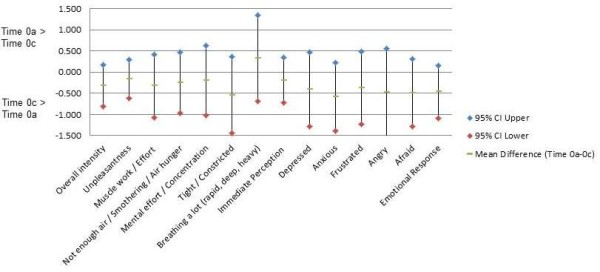
**Mean within-subjects differences (95% CI) Time 0a–0c (ED to follow-up) for individual items and subscales for Immediate Perception and Emotional Response (*n*** **= 67–68).** Time 0a: time of decision to come to ED recalled at enrollment during ED visit. Time 0c: time of decision to come to ED recalled at follow-up visit 4–6 weeks after ED visit.

For the test–retest interval between the ED visit and follow-up visit (Time 0a to 0c; Figure 2) mean differences for individual items ranged from −0.55 to +0.33 points, with all but 2 falling between −0.1 and −0.5 points. The mean differences for the two mean domain scores were approximately −0.2 points for Immediate Perception and −0.5 points for Emotional Response. All but one of the mean differences were negative, indicating that the follow-up ratings 4 to 6 weeks later were consistently higher (more severe) at Time 0c (during follow-up) than the initial ratings in the ED (Time 0a). However, the 95% CIs for the Time 0a–Time 0c differences (Figure 2) all contained 0 difference and were much wider than the 95% CIs for Time 0a–Time 0b differences (Figure 1).

Percentiles of agreement were narrower (i.e., agreement was closer) for the Immediate Perception items compared with the Emotional Response items for both recall intervals (see Additional file 1: Table A2 — Percentiles of within-subjects differences). For the Time 0a to 0b interval (during the ED visit), 80% of subjects (10^th^, 90^th^ percentiles) had scores within ± 1 point for the mean Immediate Perception domain score and within ± 2 points for the mean Emotional Response domain score. The ranges between percentiles of agreement were considerably wider for the Time 0a to 0c interval.

## Discussion

Overall, internal consistency reliability (Cronbach’s alpha) was strong for both MDP domains (Immediate Perception and Emotional Response) across all three recall administrations. During the ED visit, test–retest reliability in recall MDP ratings for dyspnea at the time participants decided to seek care in the ED was strong for individual items and very strong for the two domains (Table 3). Within-subjects agreement (intra-rater reliability) was satisfactory for both domains (Additional file 1: Table A2). In contrast, for the much longer recall interval between the ED and follow-up visits, the test–retest reliability (Table 3) and within-subjects agreement (Additional file 1: Table A2) were poor for individual items and significantly attenuated for the two domains.

For the short recall interval during the ED visit, the median within-subjects difference in scores was 0 for individual items and from 0 to 0.2 for the mean domain scores (Additional file 1: Table A2). There was a small but consistent shift toward lower ratings on the second administration in the ED (Table 1). Assuming the earliest recall rating as the reference standard, the consistency and amount of shift indicates a systematic error or bias of approximately +0.3 points on average as reflected in the positive mean within-subjects differences (Figure 1). This shift was also evident in absolute values of within-subjects differences at the 75^th^, 90^th^, and 95^th^ percentiles generally exceeding the corresponding absolute values at the 25^th^, 10^th^, and 5^th^ percentiles, respectively (Additional file 1: Table A2).

For the much longer test–retest interval between the ED and follow-up visits, median within-subjects differences were 0 for individual items and −0.2 to +0.1 for the mean domain scores (Additional file 1: Table A2). There was a small but consistent shift toward higher recall ratings at the follow-up compared with the initial recall ratings in the ED. This was reflected in the negative mean within-subjects differences of approximately −0.3 points for the Immediate Perception items and −0.5 points for the Emotional Response items (Figure 2). This shift was also evident in absolute values of within-subjects differences at the 25^th^, 10^th^, and 5^th^ percentiles generally exceeding the absolute values of differences at the 75^th^, 90^th^, and 95^th^ percentiles (Additional file 1: Table A2).

The magnitude of these shifts was small across both test–retest intervals. In addition, the 95% CI for differences for a majority of the individual items in Figure 1 (Time 0a–Time 0b) and for all individual items and domain scores in Figure 2 (Time 0a–Time 0c) are consistent with 0 difference, and the 95% CI in Figure 2 are much wider than in Figure 1. However, within each recall interval, the shifts were in same direction throughout the percentile distributions of within-subjects differences for items and domains (Additional file 1: Table A2), suggesting that the shifts are not due to outliers. In Figure 1, it is noteworthy that the point estimates for mean paired differences are > 0 for each mean domain score and for 11 of 12 individual items, whereas in Figure 2, the point estimates for mean paired differences are < 0 for each mean domain score and for 11 of 12 individual items. The consistency of those shifts within each test–retest interval is unlikely under a null hypothesis of random error around 0 difference and, on that basis, we believe systematic error (bias) to be a more plausible explanation. However, these shifts were not anticipated findings and deserve further investigation before any firm conclusions can be drawn.

We found that test–retest reliability for the items and mean domain score for Immediate Perception was stronger than for the Emotional Response items and domain score. In several studies in the pain literature, recall was more reliable and accurate for sensory compared with affective ratings [60] or pain descriptor choices [61].Although the component structure of the MDP recall ratings was similar across administrations, one notable difference was that *Frustrated* was the Emotional Response item with the strongest loading in both ED administrations, whereas *Afraid* was the strongest loading Emotional Response item during the follow-up visits (Additional file 1: Table A1).

In contrast to our findings, studies of neurological symptoms, specifically dizziness [3] and headache [4], have found substantial imprecision or lack of concordance in response to the same questions on two occasions in the ED [3] or to two semantically similar questions asked concurrently [4]. However, in both of those studies, the recall or concordance task involved nominal categories (i.e., qualitative descriptor categories [3] or dichotomous, yes/no type, choices [4]), not rating scales (as in the present study). It may well be the case for self-reported symptoms that test–retest reliability (or the assessment thereof) is facilitated if numerical rating scales are used rather than nominal (unordered) categorical choices. Alternatively, it is conceivable that symptom recall in the ED may be more reliable for dyspnea than it is for dizziness or headache.

An important limitation of the study is that we were unable to measure pre-arrival dyspnea in real time. The use of recall ratings was necessitated by limitations on approaching patients for participation until after initial clinical evaluation. In addition, the protocol did not include objective measures related to dyspnea during the ED visit against which the recall ratings could be assessed. However, in a previous publication [28] MDP “now” ratings during the follow-up visit were significantly and positively correlated with other measures of functional limitation due to breathlessness or fatigue, somatization, depression, and anxiety.

Other study limitations included convenience sampling, exclusion of patients who were unstable, and practical and ethical constraints on when initial contacts with patients and enrollment could occur relative to arrival in the ED. In addition, there were several limitations to our statistical analysis. Convenience sampling is difficult to avoid in observational studies with acutely ill patients, and we necessarily had to exclude patients who were unstable or whose capacity to consent was adversely impacted by their condition. Although participation was limited to English-speaking patients, nearly all exclusions on that basis were of patients who were Spanish speaking. Nonetheless, more than a quarter of participants were Hispanic. With respect to statistical analysis, we used principal components analysis rather than factor analysis to assess domain structure of the recall ratings. Estimates for component loadings, communalities, and total explained variance tend to be somewhat inflated for principal components compared with factor analysis. However, they generally agree on the number of components or factors to keep and which items load primarily on which factors [62-64] (see Additional file 1: *Principal components analysis* and Table A1).

At the same time, several strengths of this study are notable. Apart from the limitations noted above, our inclusion criteria were broad, and our sample was diagnostically heterogeneous, suggesting that use of the MDP in the ED is not diagnosis-specific. We believe that enhances its potential usefulness in the ED. In conjunction with previous evidence of internal validity of the MDP (e.g., that items can discriminate between different dyspnea stimuli in controlled experiments [26] and that “now” ratings are responsive to clinical change in the ED [28]), results of the present study support its external validity. In addition, as recommended by Broderick and colleagues [5], we used a multiple-item instrument, gave clear and consistent instructions as to the rating task and dimensions to be rated, and recall was referenced to a specific point in time, the decision to come to the ED. Our results demonstrate high reliability in dyspnea recall when using the MDP during an ED visit and a high degree of similarity in factorial structure to MDP “now” ratings obtained after initiation of treatment [28]. However, we also found that test–retest reliability was poor for individual items and markedly decreased for domain scores over a 4- to 6-week recall interval between the ED and follow-up visits.

## Conclusion

At a fundamental level, reliability estimates can be thought of as signal-to-noise ratios [18]. Undoubtedly, there is greater noise in symptom self-reports than in many measures of more objective data. However, at least some of the noise in symptom self-reports comes from asking noisy (e.g., ambiguous or poorly focused) questions, a problem that is potentially treatable by using a reliable and valid questionnaire such as the MDP [26-28]. Although it might seem intuitive that one should ask patients to recall pre-visit events or perceptions as soon as possible after arrival in the ED, the results of this study suggest that within the span of an ED visit, recall of dyspnea is sufficiently stable that the actual time lag between arrival and a more detailed assessment with the MDP may not be critical while the patient is in the ED and should not be viewed as a barrier to the use of this measure in the ED.

## Competing interests

MBP, PMM, DS, JA, and PB have no competing interests.

## Authors’ contributions

MBP and PMM conceived of the study and participated in all aspects of its design and coordination, and planned and conducted the statistical analysis. DS and JA participated in the design of the study protocol, data acquisition, and interpretation of results. PB participated in data acquisition and study coordination. MBP wrote the initial draft, and all authors participated in revision of the manuscript for important intellectual content. All authors read and approved the final manuscript.

## Authors’ information

MBP is Associate Professor, University of New Mexico College of Nursing. PMM is Professor, University of Colorado Denver, College of Nursing. DS is Professor, Department of Emergency Medicine and Associate Dean for Graduate Medical Education, University of New Mexico School of Medicine. JA is Associate Professor, Department of Emergency Medicine, University of New Mexico School of Medicine and Chief of the Emergency Medicine Service, Raymond G. Murphy VA Medical Center, Albuquerque, NM. PB is retired. At the time the study was conducted, she was Project Manager, University of New Mexico, College of Nursing.

## Supplementary Material

Additional file 1 **Table A1.** Principal components analysis with varimax rotation for MDP recall ratings. Table A2 Percentiles of within-subjects differences. Click here for file
