# Supplementary material for: Test–retest reliability of multidimensional dyspnea profile recall ratings in the emergency department: a prospective, longitudinal study
Source: BMC Emerg Med. 2012 May 24;12:6. doi: 10.1186/1471-227X-12-6 (PMC3464619; doi:10.1186/1471-227X-12-6)
Supplement: Additional file 1 — Table A1. Principal components analysis with varimax rotation for MDP recall ratings. Table A2 Percentiles of within-subjects differences. [file 1471-227X-12-6-S1.doc]

**Additional File 1**

**Test–retest reliability of Multidimensional Dyspnea Profile recall ratings in the emergency department: a prospective, longitudinal study**

**Mark B. Parshall, PhD, RN^1^**

**Paula M. Meek, PhD, RN^2^**

**David Sklar, MD^3^**

**Joe Alcock, MD^3,4^**

**Paula Bittner, RN, MSN^1^**

^1^ University of New Mexico College of Nursing, Albuquerque, NM

^2^ University of Colorado College of Nursing, Denver, CO

^3^ University of New Mexico School of Medicine and Department of Emergency Medicine, Albuquerque, NM

^4^ Emergency Medicine Service, Raymond G. Murphy VA Medical Center, Albuquerque, NM

Funding Source: NIH: NR010006; PI Robert B Banzett

**Methods**

*Data analysis*

*Principal components analysis*. The domain structure for MDP recall ratings was assessed with principal components analysis (PCA) using varimax rotation, which were the extraction and rotation methods used previously for “now” ratings from the same subjects . PCA yields somewhat inflated estimates for coefficients and explained variance, relative to exploratory factor analysis (EFA) extraction methods, such as principal axis or maximum likelihood (ML) factoring. However, decisions about how many components or factors to keep are generally based on initial eigenvalues, which are identical regardless of whether PCA or an EFA extraction method is used. In general, PCA and EFA results agree on the number of components / factors (i.e., domains) and which variables (items) load primarily on which component or factor .

To determine the number of components to extract, we used scree plots of eigenvalues supported by parallel analysis , a Monte Carlo simulation of eigenvalues based on random numbers. We used a parallel analysis application to generate 1,000 correlation matrix replications for 12 items at the sample sizes for Times 0a, 0b, and 0c. Scree plots and parallel analysis were consistent with retaining only two components in each time frame (Table A1).

Components / factors are uncorrelated (orthogonal) at extraction . Varimax rotation constrains them to remain uncorrelated throughout rotation. This yields a single rotated component / factor matrix in which pattern coefficients (i.e., beta-weights used to reproduce the correlation matrix and in estimation of factor scores) are also structure coefficients (the correlations of each item with each component or factor) . Varimax rotation facilitates arriving at simple structure (i.e., each variable / item has a clear primary loading on one rotated component / factor and weak loadings on any other). Because it is a constraint applied by the researcher, results of a varimax rotation are not evidence that components / factors are, in fact, uncorrelated. The direction and degree of interfactor correlation must be determined by oblique rotation or other methods .

An oblique rotation allows the components or factors to correlate through rotation and yields estimates of interfactor correlations. Oblique rotations yield separate matrices for pattern and structure coefficients. This added complexity is important if the coefficients will be used to generate factor scores (i.e., standardized linear composites in which all items contribute some weight to every component or factor). However, for questionnaires designed with clinical use in mind, it is very common to estimate domain scores as simply the sum or mean of item scores for only those items with a strong primary loading on a given component or factor. For each component or factor, this implicitly assigns a unit-weight to the variables that have their strongest salient loading there and a zero-weight to all others . Results of a PCA with varimax rotation are generally adequate for that kind of scoring .

For each recall time frame, we compared the two mean domain scores calculated from our varimax rotated PCA against factor scores estimated from EFA using ML extraction with direct oblimin rotation. Correlations between the two domain scores ranged from r = .52 to r = .59 (p < .001) and were virtually identical to interfactor correlations estimated from the oblique rotation. Correlations between the mean domain scores and their corresponding factor scores were all ≥ .98 for Immediate Perception and ≥ .96 for Emotional Response.

**Table A1 Principal components a**nalysis with varimax rotation for MDP recall ratings

|  | Time 0a (*n* = 150) | | | Time 0b (*n* = 140) | | | Time 0c (*n* = 67) | | |
| --- | --- | --- | --- | --- | --- | --- | --- | --- | --- |
|  | Component | |  | Component | |  | Component | |  |
|  | **1** | **2** | **h^2^** | **1** | **2** | **h^2^** | **1** | **2** | **h^2^** |
| Not enough / Smother / Air hunger | **0.88** | 0.16 | 0.80 | **0.88** | 0.21 | 0.82 | **0.86** | 0.22 | 0.79 |
| Muscle work / Effort | **0.85** | 0.25 | 0.79 | **0.84** | 0.31 | 0.80 | **0.89** | 0.15 | 0.82 |
| Immediate unpleasantness | **0.80** | 0.32 | 0.75 | **0.84** | 0.33 | 0.81 | **0.71** | 0.35 | 0.63 |
| Mental effort / Concentrate | **0.74** | 0.29 | 0.63 | **0.79** | 0.39 | 0.77 | **0.72** | 0.33 | 0.62 |
| Breathing a lot (rapid, deep, heavy) | **0.72** | 0.08 | 0.53 | **0.81** | 0.12 | 0.67 | **0.74** | 0.10 | 0.56 |
| Overall intensity of breathing sensation | **0.72** | 0.18 | 0.55 | **0.83** | 0.26 | 0.76 | **0.76** | 0.24 | 0.64 |
| Tight / Constricted | **0.71** | 0.16 | 0.54 | **0.79** | 0.26 | 0.69 | **0.77** | 0.16 | 0.62 |
| Frustrated | 0.23 | **0.84** | 0.76 | 0.17 | **0.88** | 0.80 | 0.28 | **0.75** | 0.63 |
| Anxious | 0.29 | **0.75** | 0.64 | 0.33 | **0.69** | 0.59 | 0.45 | **0.59** | 0.56 |
| Angry | 0.18 | **0.74** | 0.58 | 0.24 | **0.80** | 0.70 | 0.12 | **0.70** | 0.50 |
| Afraid | 0.30 | **0.72** | 0.60 | 0.39 | **0.68** | 0.62 | 0.13 | **0.87** | 0.78 |
| Depressed | 0.03 | **0.66** | 0.44 | 0.14 | **0.71** | 0.53 | 0.19 | **0.73** | 0.57 |
|  |  |  | Total |  |  | Total |  |  | Total |
| Initial eigenvalues / Extraction SS^a^ | 5.84 | 1.77 | 7.61 | 6.91 | 1.65 | 8.56 | 6.00 | 1.71 | 7.72 |
| Explained variance (%) at extraction | 48.6 | 14.8 | 63.4 | 57.6 | 13.7 | 71.3 | 50.0 | 14.3 | 64.3 |
| Rotation SS | 4.50 | 3.11 | 7.61 | 5.14 | 3.42 | 8.56 | 4.63 | 3.09 | 7.72 |
| Explained variance (%) after rotation^b^ | 37.5 | 25.9 | 63.4 | 42.8 | 28.5 | 71.3 | 38.6 | 25.7 | 64.3 |
| Cronbach’s α | .89 | .81 |  | .94 | .85 |  | .90 | .81 |  |

Component loadings in boldface type are primary loadings for each item.

Time 0a: dyspnea at time of decision to come to ED recalled at enrollment during ED visit.

Time 0b: dyspnea at time of decision to come to ED recalled 1 hr after enrollment during ED visit.

Time 0c: dyspnea at time of decision to come to ED recalled at follow-up visit 4-6 weeks after ED visit.

h^2^ = Communality estimates at extraction (proportion of variance in each item accounted for by the extracted components); SS = sum of squared component loadings; explained variance (%) = SS / (#of items) x 100.

^a^Parallel analysis threshold values for a third eigenvalue were approximately 1.26 for Times 0a and 0b and 1.37 for Time 0c, compared with observed third eigenvalues of .760, .695, and 1.04, respectively.

^b^With maximum likelihood (ML) extraction the corresponding total explained variance estimates were 56.6%, 65.8%, and 57.7% for Times 0a, 0b, and 0c, respectively. Within each component, differences in explained variance between PCA and ML extraction were all ≤ 4%.

**Table A2 Percentiles of within-subjects differences**

| Item / Domain Mean | Percentiles of Difference Times 0a minus 0b  (*n* = 142) | | | | | | | Hodges–Lehman Estimator  (95% CI) |  |
| --- | --- | --- | --- | --- | --- | --- | --- | --- | --- |
|  | 5 | 10 | 25 | 50 | 75 | 90 | 95 | For Median Difference | *P* value* |
| Sensory intensity | -2.0 | -1.0 | 0.0 | 0.0 | 0.0 | 1.0 | 3.0 | 0.0 (0.0, 0.0) | .250 |
| Unpleasantness | -1.0 | -1.0 | 0.0 | 0.0 | 1.0 | 2.0 | 2.0 | 0.0 (0.0, 0.5) | **< .001** |
| Muscle work | -2.0 | -1.0 | 0.0 | 0.0 | 1.0 | 2.0 | 4.8 | 0.0 (0.0, 0.5) | .040 |
| Not enough air | -2.0 | -1.0 | 0.0 | 0.0 | 1.0 | 2.0 | 3.0 | 0.0 (0.0, 0.5) | .021 |
| Mental effort | -2.9 | -2.0 | 0.0 | 0.0 | 1.0 | 2.0 | 3.9 | 0.0 (0.0, 0.5) | .108 |
| Tight | -2.8 | -1.0 | 0.0 | 0.0 | 1.0 | 2.0 | 3.0 | 0.0 (0.0, 0.5) | .031 |
| Breathing a lot | -3.9 | -2.0 | 0.0 | 0.0 | 1.0 | 2.0 | 4.8 | 0.0 (0.0, 0.5) | .168 |
| Immediate Perception | -1.5 | -0.9 | -0.2 | 0.0 | 0.7 | 1.4 | 2.3 | 0.14 (0.00, 0.29) | .010 |
| Depressed | -5.0 | -2.7 | 0.0 | 0.0 | 1.0 | 3.0 | 4.8 | 0.0 (0.0, 0.0) | .810 |
| Anxious | -2.9 | -2.0 | 0.0 | 0.0 | 1.0 | 3.0 | 3.9 | 0.0 (0.0, 0.5) | .040 |
| Frustrated | -3.9 | -2.0 | 0.0 | 0.0 | 1.3 | 3.0 | 5.0 | 0.5 (0.0, 1.0) | .**002** |
| Angry | -3.0 | -2.0 | 0.0 | 0.0 | 1.0 | 3.0 | 3.0 | 0.0 (0.0, 0.5) | .110 |
| Afraid | -3.0 | -1.0 | 0.0 | 0.0 | 1.0 | 2.0 | 4.0 | 0.5 (0.0, 0.5) | .008 |
| Emotional Response | -1.8 | -1.4 | -0.2 | 0.2 | 1.0 | 1.8 | 2.6 | 0.30 (0.10, 0.50) | **.003** |
|  | Percentiles of Difference Times 0a minus 0c  (*n* = 66) | | | | | | |  |  |
|  | 5 | 10 | 25 | 50 | 75 | 90 | 95 |  |  |
| Sensory intensity | -5.0 | -3.2 | -1.0 | 0.0 | 1.0 | 2.0 | 2.6 | 0.0 (-0.5, 0.5) | .395 |
| Unpleasantness | -3.0 | -3.0 | -1.0 | 0.0 | 1.0 | 2.0 | 3.2 | 0.0 (-0.5, 0.5) | .486 |
| Muscle work | -6.0 | -4.0 | -2.0 | 0.0 | 1.0 | 3.0 | 5.0 | 0.0 (-1.0, .50) | .317 |
| Not enough air | -6.0 | -5.0 | -2.0 | 0.0 | 1.0 | 3.0 | 4.6 | 0.0 (-1.0, .50) | .481 |
| Mental effort | -7.0 | -5.0 | -2.0 | 0.0 | 1.0 | 3.2 | 5.0 | 0.0 (-0.5, .50) | .717 |
| Tight | -9.6 | -6.1 | -2.0 | 0.0 | 1.0 | 4.0 | 6.1 | 0.0 (-1.0, 0.5) | .357 |
| Breathing a lot | -8.0 | -4.2 | -2.0 | 0.0 | 2.0 | 8.0 | 9.0 | 0.0 (-0.5, 1.0) | .696 |
| Immediate Perception | -4.5 | -2.8 | -1.8 | 0.1 | 1.1 | 2.4 | 3.5 | -0.14 (-0.64, 0.42) | .598 |
| Depressed | -8.0 | -6.1 | -2.0 | 0.0 | 1.0 | 5.0 | 6.0 | 0.0 (-1.0, 0.5) | .429 |
| Anxious | -7.6 | -5.2 | -2.0 | 0.0 | 1.0 | 4.0 | 5.0 | -0.5 (-1.0, 0.0) | .188 |
| Frustrated | -8.0 | -6.0 | -2.0 | 0.0 | 1.0 | 4.0 | 6.6 | 0.0 (-1.0, 0.5) | .447 |
| Angry | -9.6 | -8.2 | -1.0 | 0.0 | 1.0 | 5.0 | 8.2 | 0.0 (-1.0, 0.0) | .365 |
| Afraid | -7.0 | -6.0 | -2.0 | 0.0 | 1.0 | 3.1 | 5.0 | 0.0 (-1.0, 0.0) | .292 |
| Emotional Response | -6.2 | -3.8 | -1.8 | -0.2 | 0.8 | 2.6 | 3.6 | -0.30 (-0.90, 0.20) | .270 |

Time 0a: dyspnea at time of decision to come to ED recalled at enrollment during ED visit.

Time 0b: dyspnea at time of decision to come to ED recalled 1 hr after enrollment during ED visit.

Time 0c: dyspnea at time of decision to come to ED recalled at follow-up visit 4-6 weeks after ED visit.

*Wilcoxon signed rank test; threshold for statistical significance after Bonferroni correction *P* < .004.
